# Business Integrity

**Published:** 1870-01

**Authors:** 


### BUSINESS INTEGRITY.

There are times of weakness to us all, perhaps, when the conviction comes sweeping over us, that it is impossible to succeed in business in New York and be honest. And yet a little closer examination into the histories of men will show triumphantly that, even in New York City,

### HONESTY PAYS.

I called into the establishment on Fifth avenue the other day, where every person seemed busy in waiting upon the numerous purchasers, every one of whom seemed, by dress and manner, to be from the wealthier classes. There were packages for Germany for Egypt, for England, for Paris and other foreign countries; some were orders, others were as presents from New Yorkers whose friends were abroad. This is the most elegant establishment in the city, for it is there that

### STUART'S CANDIES

are sold in their purity. I noticed that the persons behind the counter always made the balances go down heavily, so as to give the customer the fullest weight. Just over the counter I observed a spread eagle holding in its beak a pair of scales, evenly balanced, as much as to say: There is justice done here. Good weight, pure materials, and made in the very best and most faithful manner, have given this house a world-wide reputation; and although it is the highest-priced establishment of the kind in the world, they are coining money, because everybody understands that the articles purchased are pure and good.

### A. T. STEWART

has made by his own personal energy and enterprise the largest fortune in America.

"I'M NEVER CHEATED at Stewart's," said a lady to me ten years ago, as a reason for her going there for everything she wanted in his line. And it is well known that if a clerk is found representing goods to be better than they are, he is promptly dismissed. And it has been announced as a main ambition in Mr. Stewart's life, to prove to the world that

TRUTH CAN PROSPER.

Another man, in another line of trade, has, from small beginnings in a useful and honorable business, risen to the retail of five or six thousand dollars a day, taking for his motto,

NO MISREPRESENTATION HERE.

Every man of the many busy workers in the large establishment on Broadway and Canal street, of

BALDWIN, THE CLOTHIER, is made to understand that all the work must be honestly done, and must never be represented, on pain of instant dismissal, to be what it is not; that prices are absolutely fixed, and that the terms to all, high and low, are the same for cash; for he says: "Where business is done strictly for cash, there is no risk, and the profits need not be large to insure success;" and that when goods are sold at low prices, the consumption increases; when high prices rule, sales diminish. "Patronage lost is hard to get back;" hence the best way to get custom and to keep it, is always and everywhere to

"BE HONEST."

